# Barriers and facilitators to accessing adolescents’ mental health services in Karachi: users and providers perspectives

**DOI:** 10.1186/s12913-024-10593-0

**Published:** 2024-02-01

**Authors:** Zainab Mubeen, Zafar Fatmi, Waqas Hameed, Muhammad Asim

**Affiliations:** 1 Department of Community Health Sciences, Aga Khan University, Karachi, Pakistan; 2 Centre of Excellence in Women and Child Health, Aga Khan University, Karachi, Pakistan; 3 Population Research Center, University of Texas at Austin, Austin, TX USA

**Keywords:** Mental Health, Adolescents, Barriers, Facilitators

## Abstract

**Introduction:**

Adolescents’ Mental Healthcare (MHC) is influenced by numerous factors, and adolescents occasionally seek professional help for mental health (MH) issues. These factors become more complex within low-middle-income countries (LMICs); therefore, this study aims to understand barriers and facilitators to access mental health services among adolescents aged 10 to 19 years old from the perspective of users (parents) and providers (Mental Healthcare Providers - MHPs).

**Method:**

Using a qualitative exploratory design, a semi-structured interview guide was developed using Andersen’s health service utilization model. In-depth interviews were conducted with MHPs (*n* = 21) and parents of adolescents (*n* = 19) in the psychiatry department of public and private hospitals in Karachi, from October—December 2021. Data was thematically analyzed using an inductive approach.

**Result:**

The findings revealed a consensus of users and providers in all three categories of the Andersen model and referred the compulsion as the major driving force to MHC access and utilization rather than personal choices. Within pre-disposing, need, and enabling factors; the participants highlighted a unique perspective; users regarded frequent migration, daily wage loss, and women’s societal status as barriers while the need for marriage and patient willingness were stated as facilitators. Whereas, MHPs indicated societal tolerance, the burden on the health system, and the absence of Child and Adolescent Mental Health (CAMH) services as major gaps in service delivery.

**Conclusion:**

Service utilization is mainly facilitated by the severity of illness rather than healthy choices and beliefs, and accessibility and affordability. It is therefore imperative to prioritize adolescent MH through promotion and prevention approaches and address service delivery gaps to prevent treatment delays via task-shifting and capacity building of the health workforce.

**Supplementary Information:**

The online version contains supplementary material available at 10.1186/s12913-024-10593-0.

## Background

Adolescence, a transitional phase between the ages of 10 and 19, is characterized by innumerable emotional, behavioral, hormonal, and physical changes that make an individual vulnerable to stress, aggression, risk-taking behaviors, and other psychological challenges [1]. Globally, 20% of children and adolescents suffer from psychological and developmental disorders including delayed functioning, emotional, and behavioral illnesses (attention deficit hyperactive disorder), depression, anxiety, and other intellectual disabilities [2]. The burden of mental health illnesses disturbs an individual’s biological growth, psychosocial well-being, adult life, social relationships, and educational attainments, leading to a huge burden on families as well as on the community, society, and the health system at large [3]. Approximately, 80% of people who have a mental illness and substance abuse disorder belong to LMICs, contributing 16.6% to the global burden of disease [4]. Subsequently, findings from Pakistan also demonstrate a high prevalence of behavioral disorders in adolescent males (27.3%) [1] and a strong association of anxiety (17%) and depression (21%) with age, poverty, and lower educational status [5].

In terms of care, the literature highlights that utilization of available mental healthcare (MHC) services is influenced by service inaccessibility, health beliefs, chronic illness [5], and previous experiences [6]; ultimately resulting in a treatment delay of 8–10 years [7, 8]. Several national and international studies provide evidence of barriers that impede service use, such as demographic deprivation, perceived need, inadequate knowledge, stigma [9], and cultural and traditional associations of mental illness with supernatural powers, evil eyes, or punishment [10, 11]. Moreover, self-stigmatization and social rejection over 8–12 years also interfere with MHC, in addition to parents’ attitudes [12, 13]. Countrywide, with the increasing trend in mental illness; parents, in general, are reluctant to seek care [14]. Literature is scarce in the context of exploring and defining barriers and facilitators to adolescent mental health service utilization [15]. Additionally, the endorsement and execution of Child and Adolescent Mental Health (CAMH) and the lack of pediatric mental health care community in Pakistan are impeded by MH ignorance, perceived stigma, scarcity of Mental Health Providers (MHPs), false beliefs, cultural differences, alternative healing methods, and minimum expenditures/investments [16].

The current study is in line with investigating barriers and facilitators towards MHC utilization for adolescents in Karachi, Pakistan, since previous international studies did not include the perspective of parents and health service providers in the respective domain. Hence, the results cannot be generalized because of the distinctive demographic and socio-cultural environment of Pakistan, where healthcare is often neglected. Understanding parental and provider perspectives from different backgrounds, on the barriers and facilitators that influence access to MH services, can inform the development of targeted strategies, plans, and future interventions. This study may contribute to defining pathways for making the available MH services more accessible and acceptable, especially for adolescents to attain the quality of care required and help in making better policies and procedures. To explore the perspectives of MHPs and parents on the barriers and facilitators associated with the access and utilization of MH services for adolescents in public and private tertiary care hospitals in Karachi, Pakistan.

### Conceptual framework

**Fig. 1:**
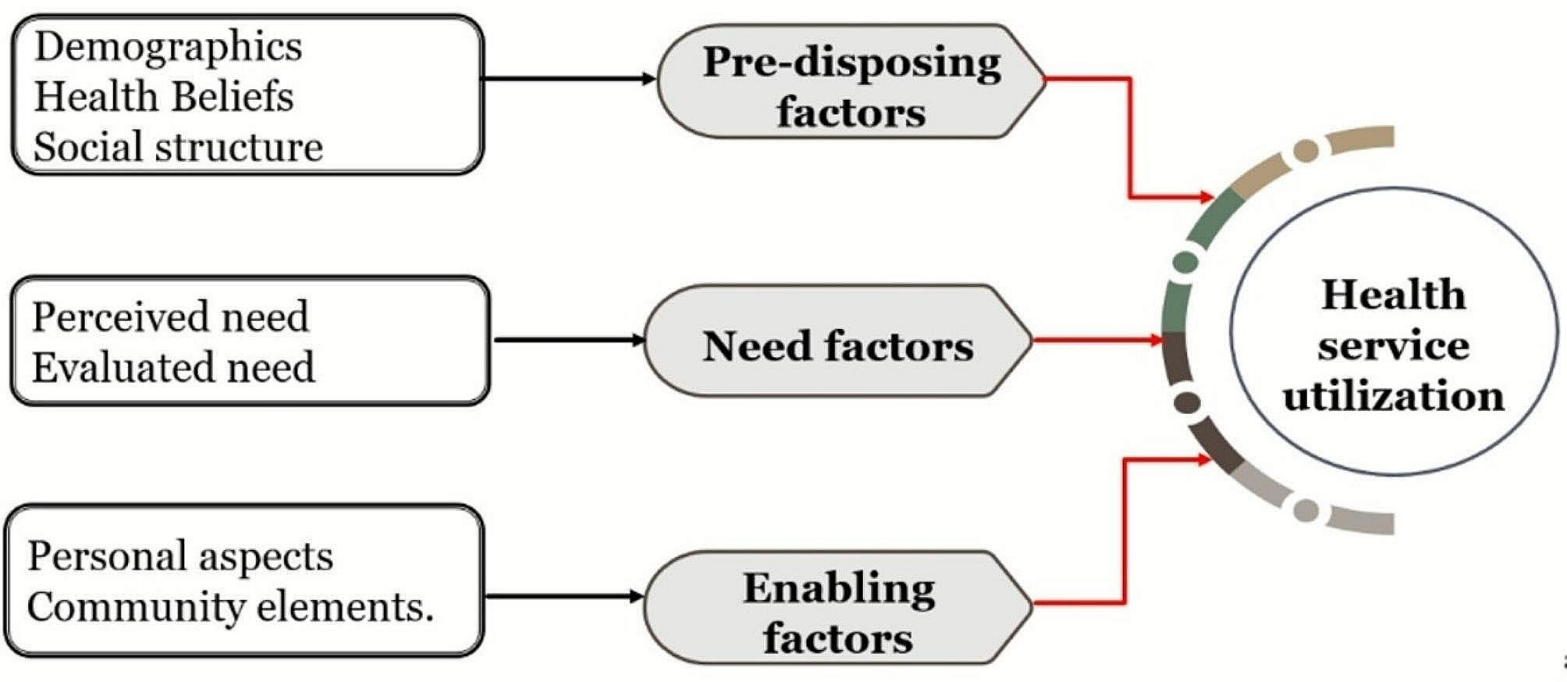
illustrates the components of Andersen’s model of healthcare utilization

This study followed the behavioral model for the identification of factors affecting health service utilization proposed by Andersen and Newman (1995), as presented in Fig. 1 - Components of Andersen’s model of healthcare utilization [17–20]. The framework emphasizes that health service utilization is affected by an individual predisposition to utilize service (predisposing factors), dynamics supporting or hindering service use (enabling factors), and elements that provoke patients to use service (need). The predisposing factors attempt to clarify an individual’s preference to seek medical help before the onset of an illness episode, such as demographics, social structure, and beliefs. Elements that expedite the fulfillment of an individual’s healthcare needs are referred to as enabling components including family characteristics and community resources. Lastly, the perceived and evaluated need for seeking medical assistance in the event of severe/chronic illness is covered within the need factors. These factors can impact healthcare utilization at both the individual and community levels [17]. The framework has been extensively employed in academic research, related to exploring the effects of psychological and socio-cultural factors (traditional norms, cultural practices, perceived need, and knowledge and attitude) in regulating the utilization of healthcare services [21, 22]. However, due to the paucity of such literature at the national level particularly for mental health services, the study aimed to employ the Andersen model to explore the predisposing, enabling, and need factors in the context of barriers and facilitators influencing the health service utilization for the mental health of adolescents.

## Methods

### Study design

The study employed a qualitative exploratory research design, which allowed the researcher to explore and describe a topic that has limited coverage within the existing literature. Further, this design enabled the study participants to contribute to the development of new knowledge in mental health service utilization for adolescents within the context of Pakistan [23].

### Sample

Using purposive sampling, a total of 40 in-depth interviews were conducted with parents [19] and MHPs [21]; recruited from psychiatric inpatient and outpatient departments of tertiary care hospitals in Karachi, Pakistan. The following inclusion criteria were used to design a pre-recruitment form and schedule interviews before formally initiating data collection.

Inclusion criteria for users and providers.

- Parents who had adolescents with any sub-category of diagnosed mental illness and receiving MH treatment from the psychiatric department.
- Parents who could communicate in English or Urdu language.
- MHPs practicing for more than three years.
- MHPs who were comfortable understanding and speaking in English and/or Urdu during the interview.

### Data collection

Based on the conceptual framework, semi-structured interview guides were developed with the collaboration of all the authors, to gather in-depth information from the participants [Supplementary File 1]. These guides underwent pilot testing and were modified accordingly, including the addition of probes to ensure comprehensive coverage of the research topics. Before conducting the interviews, informed written consent was obtained from all participants. The face-to-face interviews were conducted within the premises of the hospital, specifically in a designated noise-free area to ensure individual privacy and maintain confidentiality. The interviews were audio-recorded to ensure accurate capture of participants’ responses. The data collection period extended from October to November 2021. The study received support from the hospital administration, which facilitated the recruitment of participants and provided logistical assistance during the data collection process. The interviews focused on three key domains as outlined by the research objectives: (i) Knowledge, Beliefs, and Perceptions; (ii) Impacts of illness and driving force towards Mental Health Care (MHC); and (iii) Barriers and Facilitators related to mental health care. These domains provided a comprehensive understanding of the participants’ perspectives and experiences. Data collection continued until thematic saturation was achieved, meaning that no new significant information or themes emerged from subsequent interviews. This ensured that data saturation was reached, and further interviews were deemed unnecessary for the study’s objectives.

### Data analysis

The thematic analysis method was used to explore and code the raw data from the discussions and interviews through a deductive approach, whereas; to address the gaps within the data, the study was conducted iteratively. The transcribed information was then coded, which led to the design of sub-themes and major themes. Data were then summarized into results and findings were shared with the research team.

### Data validity

To ensure study rigor and trustworthiness, Lincoln and Guba’s four-dimensional rigorous criteria (credibility, dependability, confirmability, and transferability) were employed [24].

## Results

This section presents the findings on the barriers and facilitators to adolescents’ mental health services, from the perspectives of mental health providers (MHPs) and parents/users. The demographic characteristics of MHPs (*n* = 21) and parents (*n* = 19) who have adolescents receiving treatments from public and private tertiary care hospitals are presented in Tables 1 and 2. The study results are further presented collectively for users and providers following Andersen’s health service utilization model in Tables 3 and 4, and 5.

**Table 1:** Healthcare providers interviewed for the study

Unique codes	Domain	*N* = 21	%	Years of experience
***Psychiatrists***				
*D1, D2*	Head of the department	2	10%	More than 10 years
*CA*	Child & adolescent psychiatrist	1	5%	8 years
*F1, F2, F3, F4*	Fellows	4	19%	Up to 6 years
*R1, R2, R3, R4, R5*	Residents (Year IV)/Clinical Psychiatrists	5	24%	4 years
***Others (Psychologists and Occupational Therapists)***				
*P1-P6*	***Psychologists***	6	29%	3–6 years
*O1-O3*	***Occupational Therapists***	3	14%	3-5years

**Table 2:** Demographics of parents and their adolescents

Codes	Gender	Units	Parent Age (years)	Education	Occupation	Adolescent Disorders	Adolescent age (years)	Adolescent Gender	Family Size (# of people)
**Private Hospital**									
UA-I1	Female	Inpatient	42	Post-graduate	Employed	Major depression	18	Female	4
UA-I2	Male		51	Secondary school	Business	Substance use	16	Male	8
UA-O1	Male	Outpatient	54	Graduate	Employed	Depression	16	Female	5
UA-O2	Female		47	Graduate	House-wife	Social Anxiety	19	Female	6
UA-O3	Female		60	Secondary school	House-wife	Manic disorder	16	Female	6
UA-O4	Female		48	Postgraduate	Employed	OCD	19	Male	5
UA-05	Female		49	Graduate	House-wife	Clinical depression	16	Female	7
**Semi-Private Hospital**									
UD-O1	Female	Outpatient	42	Secondary	Housewife	Anxiety	18	Male	8
UD-O2	Female		42	Postgraduate	Housewife	Aggression and depression	15	Female & Male	5
UD-O3	female		50	Primary school	Housewife	Schizophrenia	20	Female	8
UD-O4	Female		44	Secondary school	Housewife	Behavioral disorder	11	Male	11
UD-O5	Male		60	Secondary school	Jobless	Depression	14	Female	6
UD-I1	Female	Inpatient	50	Primary school	Housewife	Substance use	18	male	12
UD-I2	Female		55	Graduate	Employed	Schizophrenia	18	female	6
**Public Hospital**									
UC-O1	Male	Outpatient	60	None	Daily wager	Conversion disorder	19	Female	13
UC-O2	Male		55	Primary	Daily wager	Bed wetting/Social anxiety	16	Male	10
UC-O3	Female		65	None	House-wife	Substance abuse	20	Male	15
UC-O4	Female		57	Primary	House-wife	Conversion disorder	19	Female	9
UC-O5	Male		58	Primary	House-wife	Hallucinations	18	Female	6

**Table 3:** Predisposing characteristics

Themes	Sub-theme	Open Codes	Barrier/Facilitator	Reported By
*Demographics*	Age	Increasing the age of a child	Facilitator	Both
	Gender	Being a female	Barrier	Provider
*Mental Health beliefs*	Knowledge	Lack of awareness/understanding	Barrier	Both
		Wrong perceptions regarding medications	Barrier	Both
	Attitude	Normalization of suffering	Barrier	Provider
		Delusions/evil eyes	Barrier	Both
		Societal tolerance	Barrier	Provider
		Blaming mothers	Barrier	Provider
		Acceptance of the child’s condition	Facilitator	Both
		Attached stigma	Barrier	Both
	Values	Negative perceptions	Barrier	Both
		Labeling of being MAD	Barrier	Both
		Lack of sensitization	Barrier	Provider
		Lack of trust in doctors	Barrier	Provider
*Social structure*	Religion	Perception of distance from religion	Barrier	Provider
		Perception of punishment from Allah	Barrier	Provider
	Education	Higher education	Facilitator	Both
		Language barrier	Barrier	User

**Table 4:** Need factors

Themes	Sub-theme	Open Codes	Barrier/Facilitator	Reported By
*Perceived need*	Severity of illness	Academic decline	Facilitator	Both
		Lack of appropriate diagnosis	Facilitator	Both
		Decline in performance	Facilitator	Both
		Compromised routine	Facilitator	Both
		Deteriorated mental health	Facilitator	Both
		Need for marriage proposals	Facilitator	User
		Disruptive routine	Facilitator	User
		Changed eating habits	Facilitator	User
		Patient willingness	Facilitator	User
		Complaints from schools	Facilitator	Both
*Evaluated need*	Perceived benefits	Medication side-effects	Barrier	Provider
	Providers’ attitude	Lack of trust in provider/perceived ineffectiveness	Barrier	Both
		Long waiting times	Barrier	Both
		Need for vaccination	Barrier	User
		Overburdened facilities/huge clientele	Barrier	Provider
		Navigation	Barrier	Both
		Criticism from health providers	Barrier	User

**Table 5:** Enabling factors

Themes	Sub-theme	Open Codes	Barrier/Facilitator	Reported By
*Personal factors*	Family dynamics	Lack of cooperation of one parent	Barrier	Both
		Family conflicts	Barrier	Both
		Family history of mental illness	Facilitator	Provider
		Joint family system	Barrier	Provider
		Co-morbidities in family	Barrier	Provider
		Working parents	Barrier	Provider
		Emotional support from household members	Facilitator	Provider
		History of illness	Facilitator	Provider
		Huge family size	Barrier	Provider
		Spousal support	Facilitator	User
		Household responsibilities	Barrier	User
		Frequent migration	Barrier	User
		Decision-making skills	Facilitator	User
	Affordability	Free of cost service/medicine	Facilitator	both
		Reduced service charges	Facilitator	both
		Loss of daily wages	Barrier	User
		Medical reimbursements	Facilitator	User
		Help from relatives	Facilitator	User
		Expensive service	Barrier	User
*Community factors*	Accessibility	Referrals from health providers	Facilitator	Both
		The poor reputation of health facility	Barrier	Provider
		Lack of child & adolescent friendly service	Barrier	User
		Positive reviews of psychiatrists	Facilitator	User
	Availability	Availability of MH facility nearby	Facilitator	Both
		Social networks/Links with people	Facilitator	Both
		Lack of assessment tools	Barrier	Provider

### Barriers and facilitators to adolescents’ mental health services

#### A. Pre-disposing factors

The findings related to individual predisposing factors associated with adolescent mental health services are presented in the below section. These results suggest that gender and age dynamics play a role in the utilization of mental health services, with potential differences in help-seeking behaviors and parental attitudes towards treatment-seeking for female patients, as reported by MHPs from private hospitals. Regarding parental attitudes towards mental healthcare seeking for female patients, MHPs highlighted gender and age dynamics as influential factors. As one health provider mentioned, “*There are chances of denial towards mental health from parents at the early age of their adolescents, this happens due to the wrong perception of normal mood and anger issues. However, as they grow up and see no improvement; parents start looking for options that can be of any type.” (P2 & O3)*.

In addition, there was a shared consensus among both users and providers that the lack of awareness served as the primary barrier in the treatment pathway, influenced by the prevalent community stigma and cynical beliefs. The existence of stigma and the fear of being judged have significant consequences, including a substantial delay in accessing mental health services and the misuse of financial resources by traditional faith healers. Such negative beliefs affect the families in general, causing them to relocate or face mockery as quoted by participants:

*“Five years, five years Madam. I had no clue what was going on. I couldn’t share it with anyone as well. I thought there was some black magic because my daughter is very pretty. Who knew that it would be a mental issue? I feel like I will die”—(UC-O5)*.

*“You know this stigma thing is so real. It is beyond any awareness, education, and access. Even in our field, it happens that people keep their issues hidden from their colleagues and family. Nobody wants to get judged.”—(F1).*

#### B. Need factors

In terms of the need or the necessity of accessing services; both parents and MHPs identified several factors. These include the advancement of the disease, the detrimental effects of mental health conditions on routine activities, declining academic performance, and disruptive household situations, all of which are considered significant facilitators. Additionally, the increasing desire and determination for personal growth among patients and referrals from healthcare providers, schoolteachers, and religious scholars are also emphasized as instigating factors for seeking treatment. *“The referrals and complaints from educational institutes are the last hit in the nail when it becomes mandatory to seek care.”—(R3)*.

For substance abuse patients, external elements often fail to start and continue the treatment process, and patients are usually driven to seek help merely based on their strong willpower. This was reiterated by participants, who shared their experiences of the child’s condition becoming critical, relating to substance use, suicide attempts, self-harm, and personal conflicts. One parent described their experience as:

*“His condition became severe, he started using wine, attempted suicide, started hitting himself, and fought with his elder brother as well. This was the last straw and I had to come and make him swear upon my life to seek help.”—(UC-O3)*.

In the stages of treatment continuation when the perceived need is diminished, the MHC is compromised due to dropouts, accounting for structural and functional challenges. These include the level of trust in service (provider, prescription, and therapy). The providers from public hospitals regarded overburdened facilities, workforce, and time shortage as the root causes of discontinuation. One of the MHPs working as a fellow shared their personal experience:

*“It sometimes happens that we ask patients to write their complaints on a note before coming next time so that we just prescribe medications. This is due to the overloaded facility where we do not have enough time to give to the patient.” (F1)*.

It is important to note here that these issues were not discussed by private providers considering the improved quality of care provided in the organization. Findings also evolved around the influence of providers’ perception and expertise on adolescents’ illness and treatment process, where parents shared their poor experience with MHPs.

*“Even some doctors judged and criticized us. Nobody has the right to judge my daughter.”—(UA-I1)*.

#### C. Enabling factors

Another significant factor that impacts mental health service utilization is financial affordability; however, it differs according to the range of care and service packages provided at the health facility. The current study included health facilities where service is provided free of cost or at nominal/reduced charges in public hospitals and excessive costs in private hospitals. This financial aspect is exemplified by a participant’s experience, as expressed.

*“We had to move to Pakistan, from Dubai after COVID-19, our lifestyle was changed 360 degrees and we were scared to even roam in Karachi. We were not able to pay the fees since my husband was fired from his job in Dubai.—(UD-I1)*.

Further, household dynamics play a significant role in creating barriers to adolescent mental health service utilization, treatment compliance, and disease resolution. These include large family size, domestic abuse, household responsibilities on mothers, joint family systems, resistance from household members, verbal abuse, teasing, and unhealthy parenting. Parents expressed their difficulties arising from household issues as: “*Even if the mother understands the problem of their children, they face difficulty to make family understood of the case.” (P4)*.

*“Emotional support from household members goes a long way, therapy is a two-way process, needs patience and a lot of emotional support.” (P5)*.

In terms of accessibility and availability, the links within the health system facilitate service access up to some extent. One of the heads of the department shared his first-hand experiences where links in the health system ease service use. *“This existence of knowledge makes people overcome the stigma. My guard asked me to help his son which prevented delay as he was aware of everything. Now imagine if we start talking about mental issues casually in society. A big burden can be reduced just through normalizing it.” (D2)*.

The managers of the public hospital highlighted several crucial structural barriers that require intervention, including the absence of empathetic providers, a scarcity of Child and Adolescent Mental Health (CAMH) therapists, and a lack of culturally appropriate assessment tools. One manager expressed concerns regarding the lack of a resolute child and adolescent psychiatry ward in the system. Consequently, there is hesitation in admitting children and adolescents to general adult wards due to the potential risk of abuse. It was emphasized that while hospital administration can ensure the behavior of their staff, they cannot guarantee the behavior of other adult psychiatric patients. The following quote underscores the urgent need to address these structural barriers to ensure the safety and appropriate care for child and adolescent patients within the healthcare system.

*“In the system, we do not have a specific child and adolescent psychiatry ward and therefore we are reluctant to admit children and adolescents in general adult wards. There are chances of abuse; I can take responsibility for my staff, but I cannot guarantee the behavior of other adult psychiatric patients.”—(R2)*.

From the identified barriers and facilitators, several gaps have been recognized, that hinder effective service delivery. These gaps include the lack of a CAMH-trained workforce, insufficient CAMH inpatient units, the absence of culturally acceptable assessment tools, overburdened facilities, limited provider attention, and a lack of mental health awareness and literacy. To address these challenges, it is imperative to implement targeted interventions such as increasing the availability of CAMH training programs, establishing dedicated CAMH inpatient units, developing culturally sensitive assessment tools, alleviating facility burdens, and promoting mental health awareness and education initiatives within the community.”

## Discussion

In-depth interviews with stakeholders of adolescent mental health revealed that the utilization of MHC is compulsion-oriented, rather than individual’s healthy choices, perceived needs and benefits, and service availability. Both parents and providers made a consensus on patient age, high educational status, and acceptance attitude as facilitators while, negative health beliefs, lack of knowledge regarding signs, symptoms, and pathways, societal stigma, religious delusions, and normalization were highlighted as prominent barriers. Interestingly, the study did not find any validity in the perceptions of gender disparity in care, contradicting previous findings where girls and more likely to identify psychological symptoms and receive mental health care compared to and boys [25, 26]. Correspondingly, the health beliefs and attitudes of any population have a significant role in impacting service use. These findings are consistent with the previous national and international literature based on LMICs, where service utilization was predominantly hindered by false illusions, stigmatizing beliefs, disgrace, and inadequate knowledge [12, 27, 28]. Other literature sources also highlight a negligible understanding of mental health issues, the relation of mental health with religion, and preference for traditional/religious/faith healers as key perceptions of the Pakistani community related to mental health/mental illness [29–31].

Among the unique insights, the providers underlined poor confidence in services, due to community tolerance. The MH service utilization has limited facilitators with most of the challenges existing within the domain of predisposing elements such as religious misbeliefs, and the concept of the evil eye and punishment. Moreover, the absence of a treatment pathway and early screening discourages service consumption, leading to the deterioration of illness, compromised routine, morbidity, mortality, and treatment delays. Within need, study participants emphasize the severity of illness as the most influential facilitator, sub-classified into disease progression and deterioration, poor academic performance, distressed household environment, suicide attempts, and compromised routine. These findings are in line with previous studies that indicate a strong association between health service utilization with the presence of severe symptoms/self-destructive attitude and comorbidities [32, 33]. A study involving 3500 general Pakistanis also corroborates the barriers to mental health care including a generalized lack of faith in psychotherapy and MHPs, previous negative experiences, religious factors, lack of perceived need, social stigma, and family restrictions [32].

Distinctively, parents from the private sector characterized school referrals as enabling factors; this highlights the importance of the positive role of the community and the need for in-school capacity building to sensitize parents, teachers, and the general community to prevent the adverse consequences of untreated illnesses. In contrast, participants from low socio-economic backgrounds mentioned the influence of adolescent marriage on the service utilization process, leading to early dropouts, relapse, and disease advancement. These findings are unique in the cultural and socioeconomic context of Pakistan and there is no published literature based on this aspect. For substance use patients, the willingness to seek health care was an important mediator, as reported by parents from both public and private facilities. This is inconsistent with previous literature and thus contributes strength to the study [34, 35]. Furthermore, participants reached a consensus on the challenges related to transportation, trust issues with service, and overburdened facilities, particularly in public-provided MHCs, as reported in previous studies [34, 36, 37]. Overall, the providers and users regarded the referrals from other health providers and informal recommendations as chief facilitators. These results are coherent with previous research among adolescents and caregivers who described networks and referrals as a driving force in service utilization [38, 39].

The proximity of mental health services was also reflected as an important feature in accessing care. However, it is important to note there were mixed opinions among participants indicating that it may not apply to all the study participants belonging to diverse backgrounds and cannot be generalized. The lack of MH clinics near the residence and the emerging cost of transport to reach health facilities are some of the well-known factors in create an obstacle and impact treatment dropouts, especially in LMICs [40–43].

Other enablers underlined by providers include the presence of emotional support, acceptance, encouragement to seek care, and a history of illness within the family, as opposed to parents/users. These findings highlight the strong influence of joint family systems and cultural norms on mental health care utilization. Provision of family support could therefore prevent delayed identification and perceived obligation to seek help from spiritual and faith healers [44, 45]. As previously reported, In LMICs such as Pakistan, India, Bangladesh, and Ethiopia; faith and religious healers are primary consultation resources for mental illness, especially among Muslims [46–50].

Correspondingly, resilience and decision-making skills are remarkable driving forces along with financial assistance to comply with health services. In opposition, the lack of affordability results in conveyance and medication costs, and loss of daily wages; suitably defined as barriers. In addition, participants regarded household and spousal support as major challenges, especially in the dynamics of joint families. Furthermore, the burden of household responsibilities and cultural obligations on women contribute to delays in seeking healthcare and impact adherence to therapy sessions and medication. It is worth noting that this factor was not highlighted in previous literature, as this study provides new insights within the context of Pakistan [34, 51, 52]. 

Users also highlighted frequent relocation as the additional impeding factor along with the availability of specialized CAMH services. In the same context, providers also mentioned the poor outlook of public hospitals, delayed appointments, time-consuming processes, the low reputation of the facility, and the absence of child and adolescent-specific inpatient wards as considerable barriers. The published work from LMICs emphasizes the same barriers and complications in the assessment and treatment process [53, 54]. These components in the context of Karachi, Pakistan could be addressed with the help of political will, research work focused on children and adolescents, development of policies specifically for this age group, appropriate funding, and introduction of child and adolescent-friendly MHC services. Another significant barrier highlighted by providers of public and private hospitals was the absence of psychological screening tools as per the culture of Pakistan. This is also presented in the literature along with a lack of appropriate skills to develop and use the assessment tools. Briefly, the service delivery is intricate because of a lack of trained personnel as well as cross-cultural tools for screening, particularly in settings with limited resources [10, 55].

Apart from the initial stage of accessing treatment, there is evidence of high rates of treatment dropouts, low adherence to treatment, and therapeutic failure, which lead to the worsening of the illness and pose challenges for parents. These challenges include dealing with the adverse effects of medications, financial constraints, societal pressures, difficulties in implementing behavioral and lifestyle changes, and ensuring a consistent supply of medications.

### Strengths and limitations

This was the primary study where the simultaneous involvement of multiple stakeholders from diverse backgrounds was ensured in public and private tertiary care hospitals, to determine distinctive views and identify service delivery gaps. The investigation remained limited to Andersen’s health service utilization framework restricted the exploration of barriers and facilitators within only predisposing, need, and enabling factors. Additionally, due to considerable time and resource constraints, policymakers, general providers, non-users, and other health care levels could not be included.

## Conclusion

Adolescents, being a highly vulnerable group of the population, are at an increased risk of developing mental illnesses, and if left undiagnosed and untreated, it can create a burden on both the families and the healthcare system. Surprisingly, this study highlighted that every parent faced challenges in utilizing mental health services, demonstrating the presence of barriers in the health system. Both users and providers made a consensus on certain facilitators, such as age, family dynamics, perceived need, and disease progression, which played a significant role in seeking care. Nevertheless, these factors were primarily driven by the severity of the disease, rather than by personal choices or predisposing characteristics, and service availability. This indicates the multiple gaps in mental health service delivery, including inadequate accessibility, availability, and utilization, particularly in low-resource settings due to the shortage of health workforce, facilities, finances, and common health beliefs.

It is therefore imperative to design and develop adolescent-friendly services that are accessible and affordable with adequate deployment of trained and qualified mental health professionals. To design generalizable and locally applicable measures in the future, large-scale prevalence studies and the effect of the variable on service utilization need to be studied with improvised research methodologies to understand the pertinent issues of child and adolescent mental health. Lastly, educational campaigns to address barriers within pre-disposing domains are critical to designing policies relevant to the prevention, promotion, treatment, and rehabilitation of MH. Future research should be focused on exploring the perspective of non-users and other health providers to determine their viewpoints. Also, intervention-based research involving the young population from the community is recommended to determine the uptake of MHC services in the absence of community and family barriers.

### Electronic supplementary material

Below is the link to the electronic supplementary material.

Supplementary Material 1

## Data Availability

The datasets generated and/or analyzed during the current study are not publicly available due to individual privacy and confidentiality but are available from the corresponding author upon reasonable request.

## Abbreviations

CAMH: Child and adolescent mental health services
ERC: Ethics Review Committee
LMICs: Lower Middle-Income Countries
MH: Mental Health
MHC: Mental Health Care
MHPs: Mental Health Providers
WHO: World Health Organization
