# Supplementary material for: Barriers and facilitators to accessing adolescents’ mental health services in Karachi: users and providers perspectives
Source: BMC Health Serv Res. 2024 Feb 1;24:157. doi: 10.1186/s12913-024-10593-0 (PMC10832177; doi:10.1186/s12913-024-10593-0)
Supplement: Supplementary file 1 — Supplementary Material 1 [file 12913_2024_10593_MOESM1_ESM.docx]

**Interview Guide – Providers**

My name is Zainab Abdul Qadir, and I am here from Aga Khan University; I am here to discuss adolescent mental health services, their utilization, and the barriers associated with it.

| **Gender:** | [ ] Male | [ ] Female | **Age in years:** ____________ |
| --- | --- | --- | --- |
| **Occupational group:** | [ ] Psychiatrist | [ ] Psychologist | [ ] Others |
|  | [ ] Consultant Psychiatrist | | |
| **Professional experience:** | [ ] 3 years | [ ] 3-10 | [ ] >10 years |

**In-depth interview guide for mental health care providers**

| 1. What do you know about mental health illness among adolescents?   *Probes:*  *importance of mental health*  *Nature, and severity of mental disorders and their needs*  *Impact on family, society, and economy.* |
| --- |
| 1. Do you think adolescents are vulnerable to mental health problems?   *Probes:*  *How is this age critical and what makes them vulnerable?*  *If not, then why?* |
| 1. What are the common mental health problems parents usually come up with for their children?   *Probes: Behavioral issues, ADHD, anxiety, depression, autism, etc.* |
| 1. What kind of other traditional service providers do parents normally go to before seeking professional help?   *Probes:*  *Pediatricians, faith healers, religious scholars, other therapists)* |
| 1. How chronic and delayed cases are when they normally seek professional help?   *Delay from onset to treatment* |
| 1. Is there any adequate mechanism in your facility that reduces the financial cost resulting from the use of medications and or therapy for such patients?   *Probes:*  *Welfare, Discount, Insurance*  *Telemedicine, Emergency Service* |
| 1. What do you think are the barriers/challenges Parents face to seek help   *Probes:*  *Please explain the challenges in detail*  *How do these difficulties impact the utilization of mental health services?*  *What is the most important barrier among all?* |
| 1. Once they reach the facility, what are the barriers they face in terms of service delivery and quality of care?   *Probes:*  *What prevents them from continuing treatment?* |
| 1. What do you think are the enabling factors that helped patients to reach you or other facilities?   *Probes:*  *Does reaching out early help in preventing the prolongation of disease?*  *What is the most important facilitator?* |
| 1. After prescribing medications or admission, do Parents follow your advice on these matters?   *Probes:*  *Routine follow-up?*  *Compliance with medications?*  *Therapy or counseling session?*  *Lifestyle modifications?* |
| 1. What changes do you feel would minimize the barriers within the current resources to increase access to the treatment?   *Probes:*  *Task shifting*  *Awareness sessions in school*  *Media involvement* |

That’s all for the interview. Thank you for your valuable input. Please be assured that all the information you provide will remain private and confidential.

Do you have any comments or questions regarding the interview?

**Interview Guide For Parents**

**Demographic survey**

| **Gender:** | [ ] Male | [ ] Female | | **Age in years:** ____________ |
| --- | --- | --- | --- | --- |
| **Age of adolescent with mental health issues (years)** | | | | |
| **Education:** | | |  | |
| **Occupation** | [ ] Housewife | [ ] Daily wager | | [ ] Others |
|  | [ ] Professional job | | | |
| **Family size** | [ ] > 3 people | [ ] 4-6 people | | [ ] >6 people |

**In-depth interview guide for parents**

| 1. What do you know about health?   *Probes: Health problems in children aged 10-19 years* |
| --- |
| 1. What do you know about mental health illness?   *Probes: Causes, impact* |
| 1. How do you think getting treatment for mental health is good?   *Probes: benefits or advantages* |
| 1. What were your fears when your child was first diagnosed with mental illness?   *Probes: Fear of society, ashamed* |
| 1. Do you think you can afford to seek help for mental illness for your child? If yes?   *Probes: loans, insurance, free service, help from relatives* |
| 1. What were the barriers that kept you from getting the help you needed?   *Probes: cost, distance, lack of awareness, expensive treatment, waiting time* |
| 1. What were the motivating factors which helped you to seek care**?**   *Probes: money, experience, chronic issues, good doctor, free medications, family support, zakat, and welfare.* |
| 1. As far as you and your child are concerned, how do you think mental illnesses affect their life? |
| 1. What was going on with your child when he/she was first diagnosed?   *Probes: severe issue, visit to faith healers/religious.* |
| 1. How serious do you think his/her condition was? |
| 1. At what stage, did you decide to bring your child to this organization to seek professional help?   *Probes: Condition deteriorated, compromised routine, etc* |
| 1. How do you think the current treatment plan helped your child? |
| 1. How much time has elapsed from the onset of symptoms to the appropriate treatment? |
| 1. What changes do you feel would minimize the challenges you faced in accessing the treatment?   *Probes: media awareness, the role of schools, and the role of religious scholars*. |

That’s all for the interview. Thank you for your valuable input. Please be assured that all the information you provide will remain private and confidential.

Do you have any comments or questions regarding the interview?
